# A karyotype comparison between two species of bordered plant bugs (Hemiptera, Heteroptera, Largidae) by conventional chromosome staining, C-banding and rDNA-FISH

**DOI:** 10.3897/CompCytogen.v11i2.11683

**Published:** 2017-04-13

**Authors:** Lucila Belén Salanitro, Anabella Cecilia Massaccesi, Santiago Urbisaglia, María José Bressa, Mónica Gabriela Chirino

**Affiliations:** 1 Laboratorio de Entomología Aplicada y Forense, Departamento de Ciencia y Tecnología, Universidad Nacional de Quilmes, Roque Sáenz Peña 352, Bernal (B1876BXD), Buenos Aires, Argentina; 2 Grupo de Citogenética de Insectos, Departamento de Ecología, Genética y Evolución, Facultad de Ciencias Exactas y Naturales, Universidad de Buenos Aires, Intendente Güiraldes 2160, Ciudad Universitaria, Ciudad Autónoma de Buenos Aires (C1428EHA), Argentina; 3 Consejo Nacional de Investigaciones Científicas y Técnicas, Godoy Cruz 2290, Ciudad Autónoma de Buenos Aires (C1425FQB), Argentina

**Keywords:** *Largus*, Heteroptera, C-banding, rDNA-FISH, holokinetic chromosomes, karyotype comparison

## Abstract

A cytogenetic characterization, including heterochromatin content, and the analysis of the location of rDNA genes, was performed in *Largus fasciatus* Blanchard, 1843 and *L. rufipennis* Laporte, 1832. Mitotic and meiotic analyses revealed the same diploid chromosome number 2n = 12 + X0/XX (male/female). Heterochromatin content, very scarce in both species, revealed C-blocks at both ends of autosomes and X chromosome. The most remarkable cytological feature observed between both species was the different chromosome position of the NORs. This analysis allowed us to use the NORs as a cytological marker because two clusters of rDNA genes are located at one end of one pair of autosomes in *L. fasciatus*, whereas a single rDNA cluster is located at one terminal region of the X chromosome in *L. rufipennis*. Taking into account our results and previous data obtained in other heteropteran species, the conventional staining, chromosome bandings, and rDNA-FISH provide important chromosome markers for cytotaxonomy, karyotype evolution, and chromosome structure and organization studies.

## Introduction

All species of Hemiptera studied so far present holokinetic chromosomes (i.e. without a primary constriction). Kinetic activity is restricted to the chromosome ends and the chromosomes can be regarded as telokinetic during male meiosis, but holokinetic activity is recognized in mitosis. Meiotic behaviour is slightly different depending on whether we are dealing with autosomal bivalents, sex chromosomes, m chromosomes or autosomal univalents. As a rule, autosomal bivalents are chiasmatic and segregate reductionally, whereas sex and m chromosomes are achiasmatic and divide equationally at first male meiotic division. Besides, sex chromosomes do not present a defined position at metaphases I and II. Several reports on C-positive heterochromatin in true bugs showed that C-bands are terminally located (Ueshima 1979, Manna 1984, Papeschi and Bressa 2006).

At present, the seven species cytogenetically studied of Largidae possess a low diploid chromosome number, ranging between 11 and 17 autosomes, a X0/XX sex chromosome system (male/female), except for one species, and a pair of m chromosomes, excluding the genus *Largus* Hahn, 1831 (Ueshima 1979, Manna 1984, Manna et al. 1985, Mola and Papeschi 1993, Bressa et al. 2005).

The genus *Largus* comprises 61 taxonomically described species and most of them are distributed in America, where its geographic distribution ranges from the north of the United States to the south of Argentina. Although they are more diverse and abundant in tropical and subtropical areas, in Argentina there are only seven species recorded (Melo and Dellapé 2013, Rosas and Brailovsky 2016). At cytogenetic level, *Largus rufipennis* Laporte, 1832 is the only species analysed to this date, using only conventional methods (Mola and Papeschi 1993, Bressa et al. 1998, 2005). It possesses a male diploid number of 2n = 13 = 12 + X0 and very large chromosomes. The partial karyotype analyses allowed detecting several Argentinean populations with different number of autosomal univalents, variable chiasma frequency, and the presence/absence of B chromosomes.

The main aim of this study was to describe the karyotype of *L. fasciatus* Blanchard, 1843 and examine the structure of its holokinetic chromosomes by means of C- banding and fluorescent *in situ* hybridization (FISH) with 18S rDNA probes. Using these data we performed a detailed comparison of the content and distribution of constitutive heterochromatin and the location of rDNA gene clusters between *L. fasciatus* and *L. rufipennis* collected from several fields in Argentina.

## Material and methods

### Insects

Adults and nymphs of *L. fasciatus* (12 males and 2 females) and *L. rufipennis* (6 males) were collected from 1995 to 2009 in several fields from Buenos Aires and Entre Ríos in Argentina (Table 1). The collected adults were taxonomically determined by María del Carmen Coscarón (Facultad de Ciencias Naturales y Museo, Universidad Nacional de La Plata) and specimens were deposited in the Museo Argentino de Ciencias Naturales Bernardino Rivadavia (MACN, Buenos Aires, Argentina).

**Table 1.** Species, locality, geographical coordinates, and number of adults’ collected and examined of *Largus* for chromosomal analyses discriminated by gender.

Species	City and Province from Argentina	Coordinates (DMS)	N° of individuals
*Largus fasciatus*	Tornquist, Buenos Aires	38°05'45"S, 62°13'25"W	11 males, 2 females
*Largus rufipennis*	Isla Martín García, Buenos Aires	34°11'03"S, 58°14'58"W	1 male
	Sierra de los Padres, Buenos Aires	37°56'50"S, 57°46'40"W	3 males
	Santa Catalina, Buenos Aires	34°46'11"S, 58°27'19"W	1 male
	Ceibas, Entre Ríos	33°30'02"S, 58°48'16"W	1 male

### Chromosome preparations

The captured specimens were swollen in freshly prepared fixative (methanol: glacial acetic acid, 3:1). In the laboratory their gonads were dissected out in 70% ethanol. Cells of gonads were dissociated in a drop of 45% acetic acid, prepared by the squash technique, and stored at -20º C until use. Chromosome preparations were removed from freezer, dehydrated in an ethanol series, and air-dried. For mitotic and meiotic analyses, the chromosome preparations were stained with 5% Giemsa solution following conventional procedures. Heterochromatin content and distribution were analysed by means of C-bands according to Papeschi (1988), and the pre-treated slides were stained with 4’6-diamidino-2-phenylindole (DAPI; Fluka BioChemika, Sigma Aldrich Production GmbH, Buchs, Switzerland) for a better resolution (Poggio et al. 2011).

### Fluorescence *in situ* hybridization

Spread chromosome preparations were made in a drop of 60% acetic acid with the help of tungsten needles and the spreading on the slide was performed using a heating plate at 45° C as described in Traut (1976). Unlabelled 18S ribosomal DNA (rDNA) probes were generated by polymerase chain reaction (PCR) using universal arthropod primers: forward 5´-CCTGAGAAACGGCTACCACATC-3´ and reverse 5´-GAGTCTCGTTCGTTATCGGA-3´ (Whiting 2002). Total genomic DNA of *Dysdercus albofasciatus* Berg, 1878, obtained by standard phenol-chloroform-isoamyl alcohol extraction, was used as a template. PCR was done following the profile described in Fuková et al. (2005). The PCR product showed a single band of about 1,000 bp on a 1% agarose gel. The band was excised from the gel and purified by using a QIAquick Gel Extraction Kit (Quiagen GmbH, Hilden, Germany). The 18S rDNA fragment was re-amplified by PCR and then labeled with biotin 14-dATP by nick translation using a BioNick Labeling System (Invitrogen, Life Technologies Inc., San Diego, CA, USA). FISH with a biotinylated 18S rDNA probe was carried out following the procedure described in Sahara et al. (1999) with several modifications described by Fuková et al. (2005) and Bressa et al. (2009).

### Microscopy, photographs and image processing

Preparations were observed under high power magnification using a Leica DMLB epifluorescence microscope equipped with a Leica DFC350 FX CCD camera and Leica IM50 software, Version 4.0 (Leica Microsystems Imaging Solutions Ltd., Cambridge, UK). Black-and-white images of chromosomes were recorded separately for each fluorescent dye with the CCD camera. Images were pseudo-coloured (light blue for DAPI and red for Cy3) and processed with Adobe Photoshop CS6 Version 6.1 (1999–2012) software.

## Results

**Figure 1.**
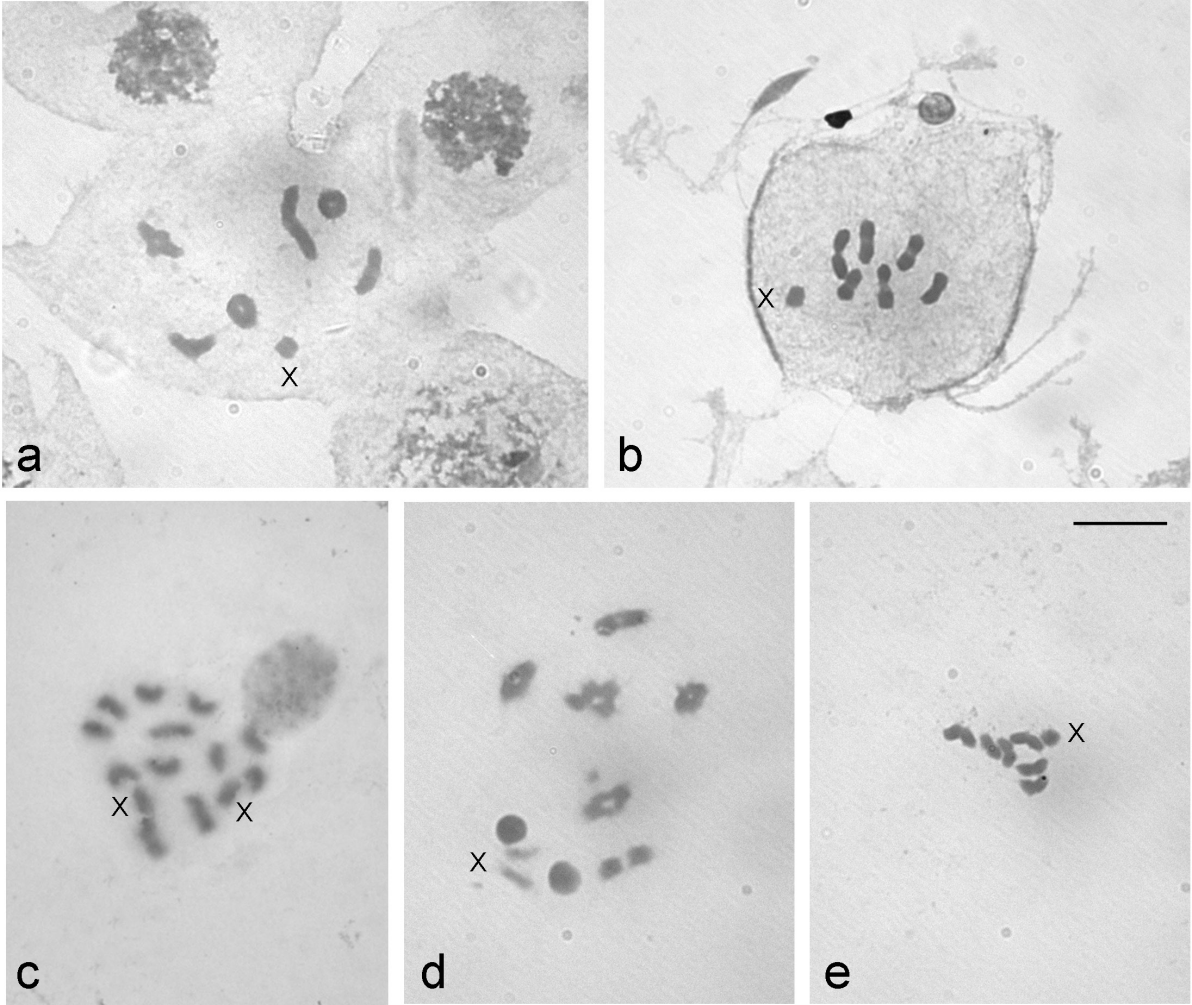
Karyotypes of *L. rufipennis* (**a–b**) and *L. fasciatus* (**c–e**) stained with 5% Giemsa. **a** diakinesis **b** metaphase I **c** oogonial metaphase (2n = 12 + XX) **d** diakinesis **e** metaphase I. X = sex chromosome. Scale bar: 10 μm.

Based on the observation of metaphase I autosomal bivalents (AA) and the identification of the sex univalent we described the male karyotype of *L. rufipennis* as 2n = 6AA + X0 (see Mola and Papeschi 1993), and the chromosome complement of *L. fasciatus* as 2n = 6AA + X0/XX (male/female sex chromosomes) (Fig. 1). In both species, the autosomes decrease gradually in size and the X chromosome is the smallest of the complement having an equal or nearly equal diameter in all directions (Fig. 1). From diakinesis onwards, the X is negatively heteropycnotic (Fig. 1a, b, d, e). The X chromosome in *L. rufipennis* is slightly longer than in *L. fasciatus* (Fig. 1b, e). At metaphases I and II, the autosomes are arranged forming a ring and with the X located outside of it (Fig. 1b–e).

**Figure 2.**
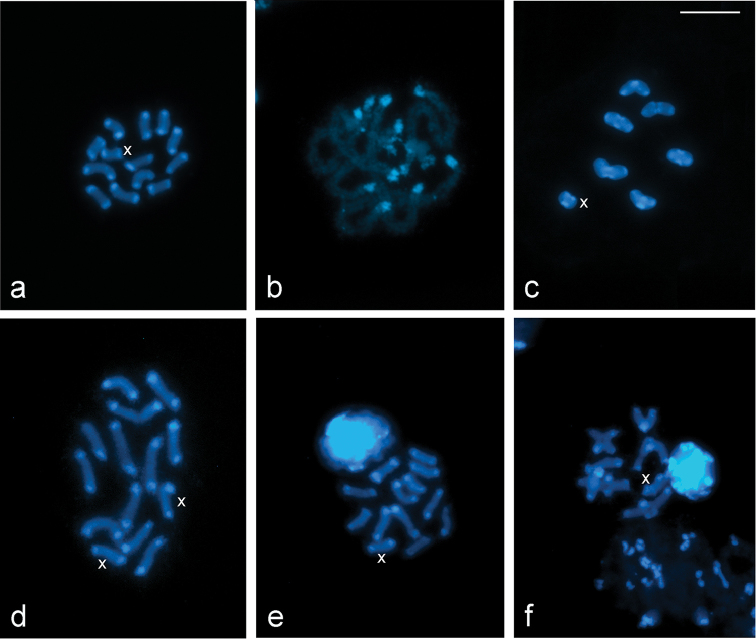
C-banding in chromosomes of *L. rufipennis* (**a–c**) and *L. fasciatus* (**d–f**) stained with DAPI. **a** spermatogonial metaphase **b** pachytene **c** metaphase I **d** oogonial promethaphase **e** spermatogonial metaphase **f** diakinesis. X = sex chromosome. Scale bar: 10 μm.

The C-banding pattern in *L. rufipennis* and *L. fasciatus* showed discrete C-positive bands terminally located in all autosomes and the X chromosomes, which were observed in all stages of mitosis and meiosis (Fig. 2).

**Figure 3.**
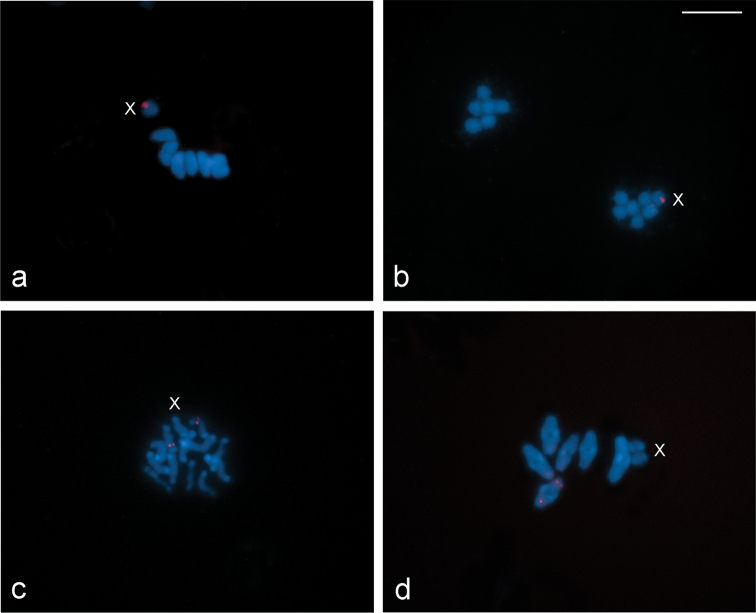
Location of rDNA genes in chromosomes of *L. rufipennis* (**a–b**) and *L. fasciatus* (**c–d**) by FISH with 18S rDNA probes (red signals). Chromosomes were counterstained with DAPI (blue). **a** metaphase I **b** telophase II **c** spermatogonial prometaphase **d** diakinesis. X = sex chromosome. Scale bar: 10 μm.

FISH experiments with the 18S rDNA probe revealed differences in the location of the probe signals between both species analysed (Fig. 3). In *L. rufipennis* a cluster of rDNA genes was located at one end of the X chromosome (Fig. 3a–b), whereas in *L. fasciatus* the hybridization signals were located at a subterminal position in an autosomal bivalent (Fig. 3c–d).

## Discussion

*Largus rufipennis* and *L. fasciatus*, the two species herein analysed, showed similar karyotypes composed of six pairs of autosomes, a simple sex chromosome system (X0/XX), and the same location and distribution of constitutive heterochromatin. The main cytogenetic difference between both species was detected in the location of the rDNA clusters. Two signals were located at a subterminal position of an autosomal bivalent of *L. fasciatus* but only one signal was observed at one end of the X chromosome of *L. rufipennis*. Taking into account the data on the chromosomal location of rDNA clusters in other heteropteran species along with our results, the NORs chromosome location varies among several congeneric species, i.e. *Belostoma* Leach, 1815 (Nepomorpha), *Triatoma* Laporte, 1832, *Panstrongylus* Berg, 1879, *Rhodnius* Stål, 1859 (Cimicomorpha), and *Dysdercus* Guérin-Méneville 1831 (Pentatomomorpha) (Papeschi and Bressa 2006, Morielle-Souza and Azeredo-Oliveira 2007, Bressa et al. 2009, Chirino et al. 2013, Pita et al. 2013, Chirino and Bressa 2014, Grozeva et al. 2014, Panzera et al. 2014), and also among different species of Tingidae (Cimicomorpha) and several species belonging to different families of Pentatomomorpha (Bardella et al. 2013, 2016, Golub et al. 2015, 2016). The analysis in a wide number of species shows that 5S, 18S, and 45S rDNA remain mainly among the autosomes, although in some species the NORs are located in the sex and m chromosomes. This might be due to the fact that NORs can be easily translocated to other chromosomes changing their number and position. Consequently, the number and location of rDNA loci (determined by FISH and/or Ag-NOR banding) constitutes an important chromosome marker, which can be useful for studies on cytotaxonomy, karyotype evolution, and chromosome structure and organization for heteropteran species. Therefore, rearrangements involving rDNA-repositioning seem to be involved in the species´ evolutionary history, indicating a particular genome dynamics for this marker.

From the cytogenetic point of view, Largidae is an interesting heteropteran family because of its low diploid chromosome number and the large chromosome size observed in most of the species (Ueshima 1979, Manna 1984, Manna et al. 1985, this study). The six karyologically analysed species of the subfamily Larginae, *Largus* and *Macrochraia* Guérin-Méneville, 1835, are characterized by the absence of an m chromosome pair, the possession of an X0/XX sex chromosome mechanism, and a number of autosomes that varies between 10 and 14. Conversely, all the studied species belonging to Physopeltinae possess 12 autosomes, two m chromosomes, and different sex chromosomes systems (X0 or X_1_X_2_Y) (see references in Papeschi and Bressa 2006). Based on the presence of a Y chromosome in very primitive heteropteran species, Nokkala and Nokkala (1983, 1984) and Grozeva and Nokkala (1996) suggested that the X0 system is a derived condition from the ancestral XY that is present in the majority of the species cytogenetically analysed. In Larginae, the X0 sex chromosome system most probably originated through the loss of the Y chromosome. The finding of a pair of m chromosomes in three species of Physopeltinae (Ueshima 1979, Manna et al. 1985) led us to suggest that this pair of chromosomes might be involved in the ancestral karyotype of the family Largidae. Then, the absence of m chromosomes and the presence of sex chromosome system X0 in species of Larginae could be considered as derived characters, which arose during karyotype evolution.

The use of different cytogenetic techniques will be very useful in further integrative studies because a group-level taxonomy followed by a reliable association among different data sets is fundamental to allow a more precise evaluation of the processes involved in the karyotype evolution and the interrelationships among different species.

## Conclusions

Taking into account the data on the number and location of rDNA clusters in *L. rufipennis* and *L. fasciatus*, we can observe two different patterns of rDNA distribution. As a result, the rDNA clusters revealed by rDNA-FISH are very useful tools for the study of the karyotype structure and chromosome evolution in groups with holokinetic chromosomes due to it can contribute to understand the karyotype evolution and taxonomic relationships among several taxa.
